# Trophic Shifts of a Generalist Consumer in Response to Resource Pulses

**DOI:** 10.1371/journal.pone.0017970

**Published:** 2011-03-18

**Authors:** Pei-Jen L. Shaner, Stephen A. Macko

**Affiliations:** 1 Department of Life Science, National Taiwan Normal University, Taipei, Taiwan; 2 Department of Environmental Sciences, University of Virginia, Charlottesville, Virginia, United States of America; Duke University, United States of America

## Abstract

Trophic shifts of generalist consumers can have broad food-web and biodiversity consequences through altered trophic flows and vertical diversity. Previous studies have used trophic shifts as indicators of food-web responses to perturbations, such as species invasion, and spatial or temporal subsidies. Resource pulses, as a form of temporal subsidies, have been found to be quite common among various ecosystems, affecting organisms at multiple trophic levels. Although diet switching of generalist consumers in response to resource pulses is well documented, few studies have examined if the switch involves trophic shifts, and if so, the directions and magnitudes of the shifts. In this study, we used stable carbon and nitrogen isotopes with a Bayesian multi-source mixing model to estimate proportional contributions of three trophic groups (i.e. producer, consumer, and fungus-detritivore) to the diets of the White-footed mouse (*Peromyscus leucopus*) receiving an artificial seed pulse or a naturally-occurring cicadas pulse. Our results demonstrated that resource pulses can drive trophic shifts in the mice. Specifically, the producer contribution to the mouse diets was increased by 32% with the seed pulse at both sites examined. The consumer contribution to the mouse diets was also increased by 29% with the cicadas pulse in one of the two grids examined. However, the pattern was reversed in the second grid, with a 13% decrease in the consumer contribution with the cicadas pulse. These findings suggest that generalist consumers may play different functional roles in food webs under perturbations of resource pulses. This study provides one of the few highly quantitative descriptions on dietary and trophic shifts of a key consumer in forest food webs, which may help future studies to form specific predictions on changes in trophic interactions following resource pulses.

## Introduction

Food-web structure can strongly influence the functioning and diversity of communities and ecosystems [1]–[3]. Generalist consumers, particularly multi-trophic omnivores, can alter trophic flows through diet switching, and therefore have long been recognized as a dynamically important feature of food webs [4]. In fact, many studies used trophic shifts, diet switching across trophic levels, as indicators of food-web responses to perturbations, such as the invasion of exotic species, and spatial or temporal subsidies to a system [5]–[6]. As a form of temporal subsidies, a resource pulse can have broad effects on species interactions [7]–[8]. Because generalist consumers are often opportunistic foragers, they are expected to respond quickly to resource pulses, both numerically and functionally [9]. However, with a decreased availability of the resource over time, and an increased consumer population, diet switching to alternative prey by the consumer often occurs, resulting in complex trophic interactions [7], [10]. In addition, the numerical responses of the generalist consumers may be so strong that they themselves become a secondary resource pulse, creating cascading effects [11]. Therefore, investigating different aspects of responses from generalist consumers to resource pulses is critical to our overall understanding of community ecology.

Although diet switching between a pulsed resource and an alternative prey has been documented for many consumer species [7], few studies have focused on trophic shifts. Therefore, it is not always clear under what circumstances diet switching will result in a trophic shift. For example, Wise et al. [12] reported that while one generalist consumer (i.e. wolf spider *Schizocosa*) shifted from herbivory to detritivory in response to a detrital subsidy, another generalist consumer (i.e. wolf spider *Hogna*) did not. Even though the mechanisms behind these species-specific responses are not yet clear, this study highlights the complexity of trophic shifts among generalist consumers receiving resource subsidies. Furthermore, resources can vary in their abundance and nutritional quality. Consequently, different types of resource pulses may have different effects on the same consumer species [13]. For example, the two classical resource pulse events, seed mast and cicadas emergence, provide two resources of very different abundance and quality. Seeds are less perishable (i.e. higher cumulative abundance) and generally have higher C:N ratios (i.e. lower nutritional quality), whereas cicadas are highly ephemeral but their soft tissues generally have lower C:N ratios. The extreme abundance of seeds might be able to drive a consumer to shift towards feeding on the producer trophic level at such a degree that would not otherwise be found under normal circumstances owing to the lower nutritional quality of plant foods. On the other hand, the ephemeral cicadas might not produce large trophic shifts in the consumer despite a higher nutritional quality. The opposite could be true as well when the nutrient constraints in the consumer [14]–[17] outweigh the energy benefits from the abundant yet less nutritious seeds, limiting the magnitudes of consumer trophic shifts. Seeds and cicadas are resources from two different trophic levels. Therefore, a diet switching to seeds or cicadas should result in a trophic shift towards the producer or consumer trophic level, respectively. However, because many consumers of resource pulses (e.g. rodents, birds) exhibit complex foraging behaviors, their diet switching to the pulsed resources may be coupled with other dietary changes, making it difficult to assume that a trophic shift will always occur. For example, if the consumers are balancing nutrient and energy requirements, they may increase their consumption of the pulsed seeds while decrease their consumption of other plant foods. As a result, their overall consumption of plant foods and their trophic role in the food web remain unchanged.

In order to examine trophic shifts, relative contributions of different trophic groups to the consumer diets must be quantified. For the past twenty years, stable isotopes have become one of the standard methods in studying animal diets [18]. A particularly useful application of stable isotopes is to estimate source contributions to the animal diets by methods of mixing models [19]–[20]. In this study, we used stable carbon and nitrogen isotopes with a Bayesian multi-source mixing model [21]–[24] to estimate proportional contributions of different trophic groups to the diets of the White-footed mouse (*Peromyscus leucopus*) receiving either an artificial seed pulse or a naturally-occurring cicadas pulse. The White-footed mouse is omnivorous, feeding on a variety of food items such as foliage, seeds, fungi, and arthropods [25]–[27]. Field experiments have shown that this species can detect relative availability of food resources with different nutritional values, and adjust their foraging effort accordingly [28]. More importantly, Peromyscus spp. have a wide geographic distribution, overlapping with several resource pulses in North America (e.g. acorn mast, cicadas emergence, El Niño rainfall events), and they have been reported to respond both numerically and functionally to resource pulses [6], [29]–[36]. Therefore, the White-footed mouse is an excellent model species for this study.

The goal of this study is to test if there are trophic shifts in the White-footed mouse in response to seed and cicadas pulses, and if so, the directions and magnitudes of the shifts. The focus is on the broad patterns of trophic shifts, looking at specifically three trophic groups: (1) producers, or plants, (2) consumers, or above-ground arthropods, and (3) fungi/detritivores. Trophic shifts of the mice were quantified by the differences in the proportional contributions of each trophic group to the mouse diets between populations that received resource pulses and those that did not.

## Methods

### Study site

The study site is at the Blandy Experimental Farm, University of Virginia, Clarke County, Virginia (78^o^00′ W, 39^o^00′ N). The region can be described as an agro-ecosystem consisting of forests and old-fields. The forests are composed of second-growth, oak-hickory (*Quercus*-*Carya*) community. The geographic area where the study site is located experiences several different types of resource pulse events, such as mast productions of acorns (i.e. seeds of *Quercus* spp.) and periodic outbreaks of the 17-year cicadas (*Magicicada* spp.). The White-footed mouse is one of the most dominant rodent species at the study site.

### Seed mast experiment

The seed mast experiment was carried out in a non-mast year (2003) at two forest sites, approximately 2 km from each other. Two 0.25-ha grids, 90 meters apart, in each of the two forest sites were included. In August of 2003, approximately 22.7 kg of millet seeds (i.e. seeds of *Panicum miliaceum*) were added on four occasions to one of the two grids in each of the two forest sites (the seed addition grids) every 10 days, resulting in a total seed density of 364 kg/ha. The cumulative seed density used here was based on a long-term (1986–1996) acorn production study at the sites within 60 km from current study site, which suggested that a density greater than 300 kg/ha represents a typical mast year [32]. The timing of the seed addition was matched to that of an acorn mast event, beginning in August and continuing through September. Millet seeds were distributed uniformly over the entire grids using a hand-held seed spreader. The remaining grid in each of the two forest sites did not receive seed supplementation (the control grids). Mice were trapped in October of 2003, and their blood samples were taken via retro-orbital bleeding for stable isotope analysis. All trapping and sampling procedures were approved by University of Virginia's Animal Care and Use Committee (Permit Number: ACUC #3021).

### Cicadas emergence experiment

During May and June of 2004, an outbreak of the 17-year cicadas occurred at the study site. Two 0.25-ha grids, 90 meters apart, in a forest site where cicadas were found to be highly abundant were included in this experiment (the cicadas emergence grids). For controls, we used data for the same grids sampled during May and June of 2003, one year prior to the emergence (the control grids). It is difficult to design a control for a cicadas emergence event because sites with and without cicadas are spatially confounded, and could not be selected at random. As a result, the differences observed could be a reflection of the sites rather than the treatment [13]. Ideally, both sites without cicadas during the emergence and sites with cicadas prior to or after the emergence should be used as controls. However, due to logistic constraints, we used the site with cicadas during a non-emergence year as the control. Mice were trapped and their blood samples taken in both years following the same protocol as in the seed mast experiment. The forest site included in this experiment was the same as one of the two sites used in the seed mast experiment. Approximately 40% of the mice from the control grids in this experiment were later re-captured in October and used in the seed experiment. However, given that fact that stable carbon and nitrogen isotopes of mouse blood tissues typically turn over in less than four months [37], the isotope values of the same mice captured four months apart between the cicadas and seed experiments were independent from each other. The trapping and sampling of the mice in the 2003 non-emergence year were completed prior to the beginning of the seed supplementation. Therefore, data for the control grids in this experiment was not affected by the seed supplementation.

### Stable isotope analysis

Potential food items for the mice, including plants, arthropods, and fungi, were collected from the study site in the summers of 2003 and 2004. Plant foliar samples included most of the common species at the study site, such as oak (*Quercus* spp.), hickory (*Carya* spp.), northern hackberry (*Celtis occidentalis*), honeysuckle (*Lonicera* spp.), and knotweed (*Polygonum* L). Ground-dwelling arthropods were collected with unbaited pitfall traps (5–8 traps per grid). In order to avoid potential sample contamination in the isotope analysis on arthropod samples, the traps had only water in them and the samples were collected daily. Moths (Lepidoptera) were collected separately at four random locations (one location per grid) using light traps. The most common arthropods captured at the study site included spiders (Araneae), harvestmen (Opiliones), beetles (Coleoptera), and moths, all of which are known prey to the mice. In addition to the plants, arthropods, and fungi, we also collected millet seeds and cicadas for isotope analysis. Considering that cicadas exoskeleton are mostly indigestible to the mice, we dissected fresh cicadas bodies and used only the soft tissues for analysis.

Two types of mouse tissues were used for stable isotope analysis, whole blood and plasma. Previous laboratory studies on mice (*Mus musculus*) have shown that the time it takes for whole blood tissues to reach isotopic equilibrium with dietary sources were approximately 70 and 112 days for carbon and nitrogen, respectively [37]. Although there are no laboratory experiments available at this time on the turnover of mouse plasma tissues, it has been shown that whole blood tissues have a longer carbon isotopic turnover time than plasma in birds (several weeks for whole blood; a few days for plasma) [38]. Therefore, in order to capture the effects of the highly ephemeral cicadas resource, mouse plasma tissues were separated from whole blood by centrifugation (at 1,200 g for 10 minutes) for isotope analysis for the cicadas grids. For the control grids in both experiments, as well as the seed grids in the seed mast experiment, whole blood tissues were used.

All samples were kept frozen prior to the drying process. Samples were oven-dried at 60^o^ for 24 to 72 hours. Approximately 1.5 mg of plant samples and 0.5 mg of animal samples were used in stable isotope analysis. Stable carbon and nitrogen isotope values (δ^13^C, δ^15^N) of the samples were measured on a Micromass Optima Isotope Ratio Mass Spectrometer connected to an elemental analyzer (EA) (GV/Micromass; Manchester, UK). Stable isotope values are reported in the conventional form:
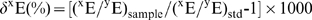
where E is the element being measured, (^x^E/^y^E)_sample_ is the isotopic composition of the sample, and (^x^E/^y^E)_std_ is the isotopic composition of a standard material specific to the element. The standard for stable carbon and nitrogen isotopic composition is Peedee Belemnite (PDB) and atmosphere molecular nitrogen (N2), respectively.

Elemental concentrations (%C, %N) of the samples were determined simultaneously with stable isotopic compositions. Molar C:N ratios of the samples were calculated based on the elemental concentrations and sample weights, and were used as indicators of the nutritional quality of the foods.

### Bayesian multi-source isotope mixing model

**Figure 1:**
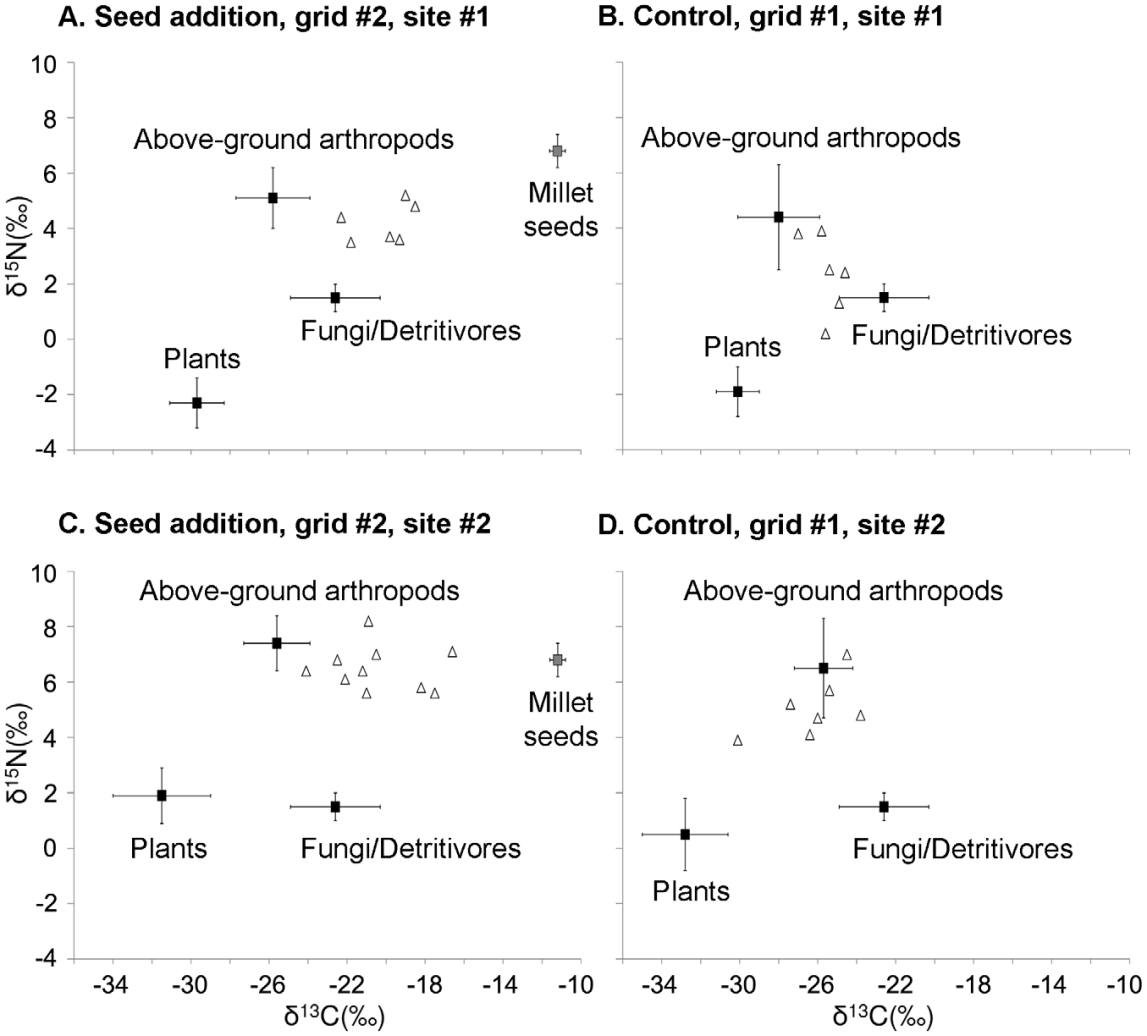
The isotope values of the dietary sources and mice in the seed mast experiment. The squares (means) and whiskers (s.d.) denote grid-specific isotope values of the dietary sources with the exception of the fungus-detritivore group, for which generic values were used. The triangles denote the isotope values of individual mice. The variability of source isotope values was incorporated into the Bayesian model using s.d. Because the model also incorporates the variability associated with discrimination factors, the values for the mice plotted here were not pre-adjusted for trophic enrichment.

**Figure 2:**
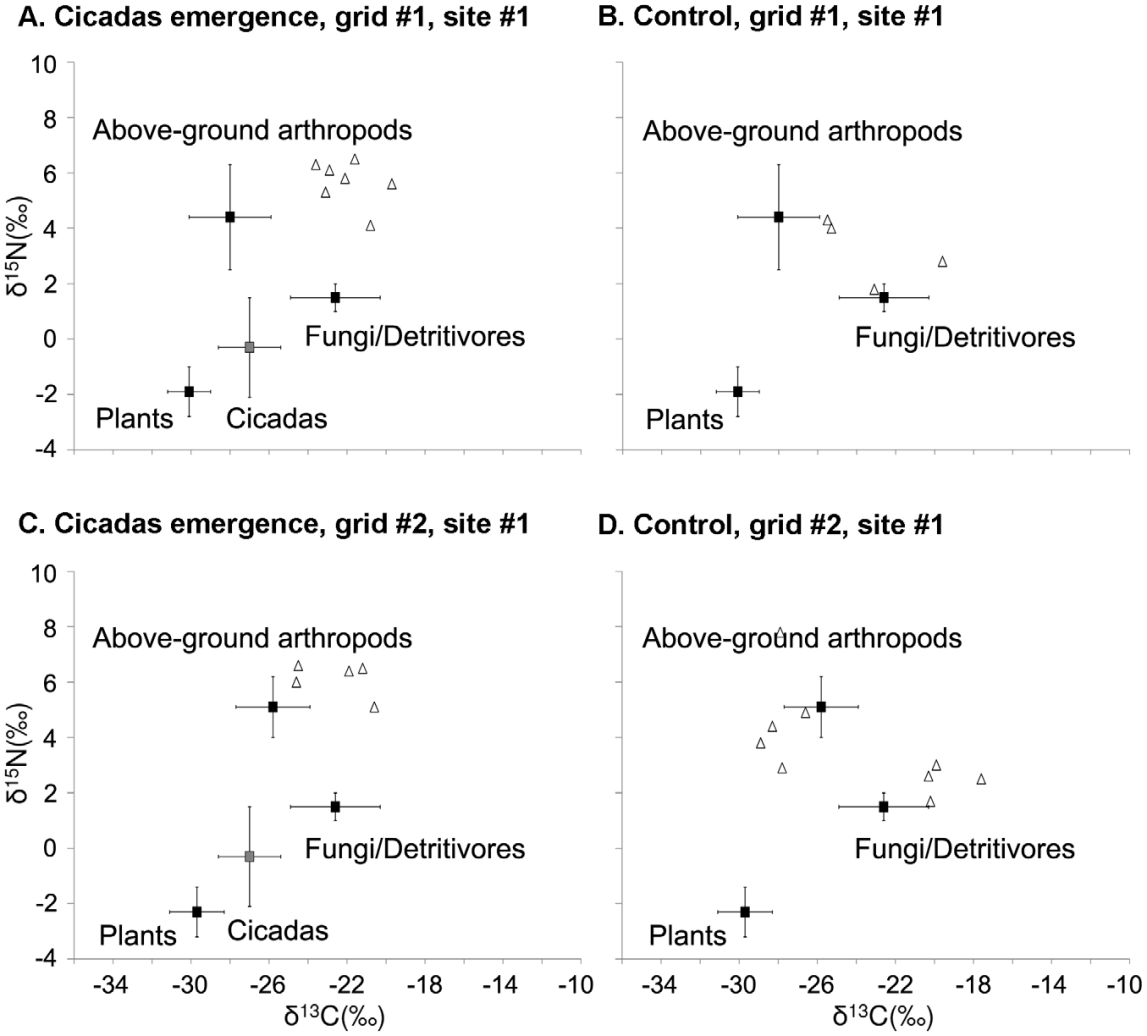
The isotope values of the dietary sources and mice in the cicadas emergence experiment. The squares (means) and whiskers (s.d.) denote grid-specific isotope values of the dietary sources with the exception of the fungus-detritivore group, for which generic values were used. The triangles denote the isotope values of individual mice. The variability of source isotope values was incorporated into the Bayesian model using s.d. Because the model also incorporates the variability associated with discrimination factors, the values for the mice plotted here were not pre-adjusted for trophic enrichment. Note that grid #1 and grid #2 were both repeatedly sampled in 2003 (used as controls) and 2004 (used as cicadas treatments). Therefore, the isotope values of the dietary sources were the same between A and B (grid #1), and between C and D (grid #2).

The proportional contribution of each dietary source to the mouse diets was estimated using software package SIAR (Stable Isotope Analysis in R) [21]. We chose to use the Bayesian approach as implemented in SIAR over linear mixing models (e.g. IsoSource by Phillips and Gregg [39]) because it incorporates the elemental concentrations, as well as the variability associated with source isotope values and discrimination factors, in the mixing model. A recent study on seabird diets suggested that a Bayesian approach could produce more robust estimates than linear mixing models [23]. However, the predictions from the Bayesian mixing model deteriorate as the number of sources is increased. Therefore, the maximum number of dietary sources was set to four in this study, which was reported to have an acceptable performance in a simulation [22]. This decision resulted in the following groupings of dietary sources: (1) for control grids in both seed and cicadas experiments, three dietary sources were included: plants, above-ground arthropods, and fungi/detritivores; (2) for seed addition grids, four dietary sources were included: plants, above-ground arthropods, fungi/detritivores, and millet seeds; (3) for cicadas emergence grids, four dietary sources were included: plants, above-ground arthropods, fungi/detritivores, and cicadas. Although fungi and detritivores are traditionally placed at different trophic levels, we grouped them into one dietary source in this study. One of the reasons for this grouping is to help control the maximum number of dietary sources used in the model. Another concern is that the isotope values of the fungus and detritivore samples at the study site were similar to each other, especially compared to the plants and above-ground arthropods (Table S1), which may not have sufficient resolution to produce a separate estimate for each of them. In order to produce robust estimates from a multi-source mixing model, it is important to reduce within-group variability in source isotope values. Therefore, grouping food items based on their similarities in the isotope values could be beneficial. However, when using these estimates to answer specific ecological questions, some combinations of the original dietary sources may be needed. The focus of this study is to detect dietary shifts of the mice among foods from the producer (e.g. plants, millet seeds), consumer (e.g. above-ground arthropods, cicadas), and fungus-detritivore (e.g. fungi, detritivores) trophic groups. Therefore, the posterior probability distributions of the plants and millet seeds were combined to represent the producer contributions in the seed addition grids, and the posterior probability distributions of above-ground arthropods and the cicadas (an herbivore) were combined to represent the consumer contributions in the cicadas emergence grids. The isotope values of the dietary sources were grid-specific, with the exception of the fungus-detritivore group. Because fungus samples were only available from one grid, and detritivore samples from two grids at the same site (Table S1), we applied the isotope values of the fungus and detritivore samples to all grids (Fig. 1 & 2). By doing so, we made the assumption that the differences in the isotope values of the fungus-detritivore groups between grids and sites are relatively small comparing to the differences among different trophic groups within the same grid.

In order to correct for trophic fractionation, we applied a discrimination factor of 0.6‰ (s.d. = 0.4‰) for carbon and 3.9‰ (s.d. = 0.9‰) for nitrogen to the δ^13^C and δ^15^N values of mouse plasma, and a discrimination factor of 0.2‰ (s.d. = 0.5‰) for carbon and 2.0‰ (s.d. = 0.9‰) for nitrogen to the δ^13^C and δ^15^N values of mouse blood tissues [40]. The discrimination factors used here are slightly different from the widely-applied values of 1‰ for δ^13^C and 3‰ for δ^15^N. This is because different tissues may have different discrimination factors [41], and tissue-specific values should always be used in favor of generic values. There are several laboratory studies available on blood discrimination factors for rodents [40], [42], and at least one of them reported plasma discrimination factors [40]. However, because lipid extraction is likely to increase tissue isotope values [43]–[45], measurements on lipid extracted blood tend to give higher discrimination factors (e.g. 1.2–2.4‰ for δ^13^C and 2.9–3.3‰ for δ^15^N in MacAvoy et al. [42]) than measurements on non-lipid extracted blood (e.g. 0.2‰ for δ^13^C and 2.0‰ for δ^15^N in Yoneyama et al. [40]). Because of the small size of the blood samples used in this study, they were not lipid extracted, as commonly done for mammalian predators [44], [46]. Therefore, we applied the discrimination factors from laboratory experiments that did not report using lipid extraction [40]. The discrimination factors were held constant for all dietary sources.

### Statistical analysis

The C:N ratios of the millet seeds and cicadas, as well as background plants and above-ground arthropods, were compared using the Kruskal-Wallis test. The δ^13^C and δ^15^N values of the mice were compared between the seed and control grids, and between the cicadas and control grids, using the analyses of variance (ANOVAs). Because the two pairs of seed addition/control grids were located at two different sites, we also included the sites as blocks in the ANOVA analysis for the seed mast experiment. The proportional contribution of each trophic group to the mouse diets is in the form of a probability distribution, containing information on which parameter values are more likely than others. Therefore, a measure of central tendency was recommended as a potential summary value that could be used for comparison purpose, particularly if the distributions are not highly skewed [22]. For descriptive and diagnosis purposes, we report the mean, median, and mode, as well as the 25–75th and 5–95th percentiles, of the probability distribution for the contribution of each trophic group to the mouse diets. Because all three summary values (mean, median and mode) suggested similar patterns, we reported the medians in all subsequent comparisons. All statistical analyses were performed in SAS 9.2.

## Results

### Nutrient quality of the resource pulses

Millet seeds had a significantly higher C:N ratio than the cicadas (Kruskal-Wallis test, *χ* ^2^ = 7.00, *P*<0.01). Similarly, the plants had a higher C:N ratio than above-ground arthropods (*χ* ^2^ = 19.87, *P*<0.0001). The C:N ratio of the millet seeds was four times higher than that of the cicadas (mean ± standard error: C:N_millet_  = 28.06±3.27, C:N_cicadas_  = 6.21±0.50), and the C:N ratio of the plants was three times higher than that of the arthropods (C:N_plants_  = 18.93±1.58, C:N_above-ground arthropods_  = 5.24±0.35). These results indicate that millet seeds were of lower nutritional values to the mice than cicadas, and this difference in nutrient quality is characteristic of that between plant and animal foods in the study system.

### Stable isotope values of the dietary sources and mice

The δ^13^C values of the plant, above-ground arthropods, and fungus-detritivore groups indicate that the food webs at the study site had a C3-based carbon source (Table S2). The δ^15^N values of the plant, above-ground arthropods, and fungus-detritivore groups showed predictable effects of trophic enrichment (Table S2). The above-ground arthropods and fungi/detritivores were approximately 6–7‰ and 0–4‰ enriched relative to the plants. Detritivores are known to have a smaller discrimination factor for δ^15^N than herbivores, omnivores, or predators [41], which explains their lower δ^15^N values. Millet is a C4 plant, and therefore had a higher δ^13^C value [47]. In addition, the millet seeds also had a higher δ^15^N value atypical of a plant, possibly a result of fertilizer influence. The cicadas had δ^13^C and δ^15^N values typical of a C3-based herbivore.

The δ^13^C and δ^15^N values of the mice were significantly higher in the seed addition grids than in the control grids (ANOVA, seed addition effect, δ^13^C: *F*_1,26_  = 63.80, *P*<0.0001, δ^15^N: *F*_1,26_ = 18.86, *P* = 0.0002; site effect, δ^13^C: *F*_1,26_ = 0.49, *P* = 0.49, δ^15^N: *F*_1,26_ = 43.94, *P*<0.0001; Fig. 1), suggesting a strong influence of the millet seeds. Because the isotope values of the cicadas were not as unique relative to other dietary sources as the millet seeds (Fig. 1 & 2), their potential influence on the mouse diets was more difficult to assess by simply comparing the isotope values of the mice. In fact, after the differences in tissue-specific discrimination factors were accounted for (i.e. the plasma tissues used for the cicadas grids were on average 0.4‰ and 1.9‰ more enriched in δ^13^C and δ^15^N than the whole blood tissues used for the control grids), the δ^13^C and δ^15^N values of the mice were not different between the cicadas and control grids (δ^13^C: *F*_1,23_ = 1.14, *P* = 0.3; δ^15^N: *F*_1,23_ = 0.56, *P* = 0.5; Fig. 2).

### Proportional contributions of different trophic groups to the mouse diets

**Figure 3:**
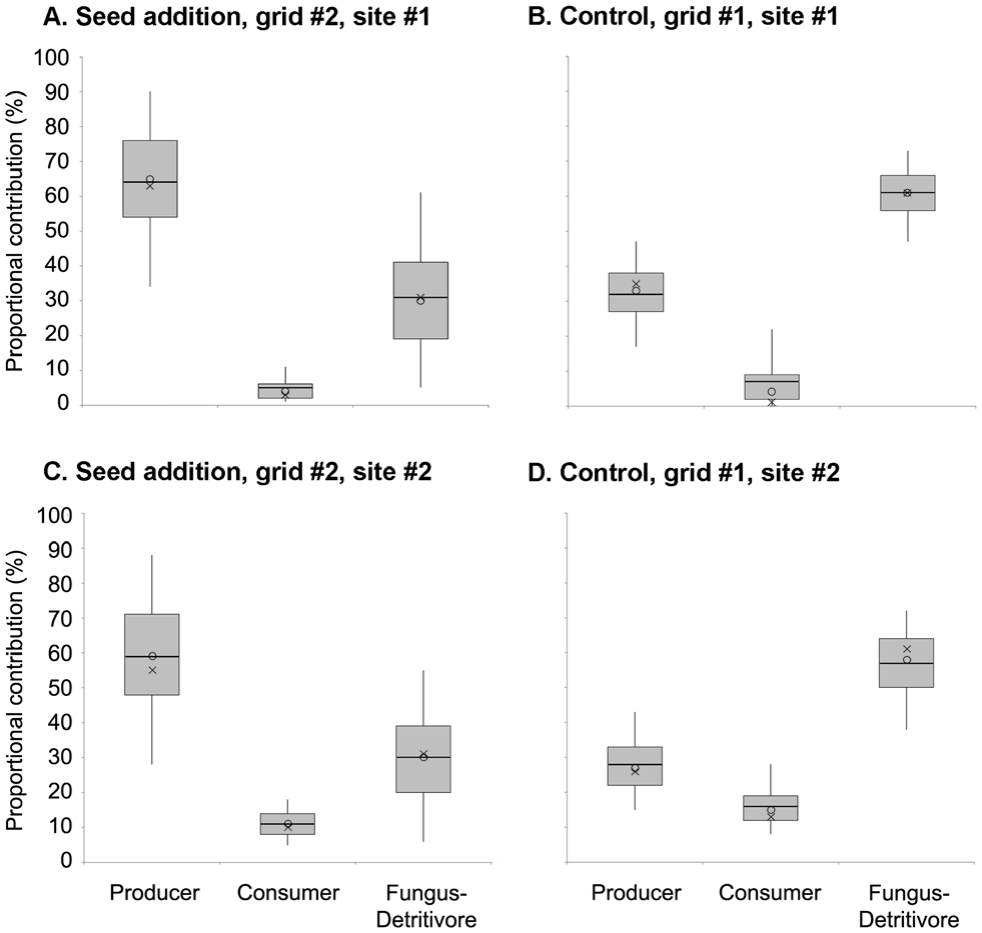
The producer, consumer, and fungus-detritivore contributions to the mouse diets in the seed mast experiment. The seed addition grids were plotted on the left and the control grids on the right for the first site (A, B) and the second site (C, D). The probability distributions of the plants and millet seeds were combined to represent the producer contributions in the seed addition grids (A, C). The lines, circles, and crosses denote the means, medians, and modes. The boxes and whiskers denote the 25–75th and 5–95th percentiles, respectively.

In the seed mast experiment, the producer trophic group contributed 32% more to the mouse diets in the seed addition grids (Fig. 3). The producer was also the most important dietary source for the mice in the seed addition grids (65% in the first grid, and 59% in the second; Fig. 3A & C) whereas the fungus-detritivore group was the most important dietary source for the mice in the control grids (61% in the first grid, and 58% in the second; Fig. 3B & D). The proportional contributions of millet seeds alone were 35% (5–95th percentiles  = 21–47%) in the first grid and 33% (5–95th percentiles  = 20–46%) in the second grid, indicating that the mice did not decrease their consumption of other plant foods as they fed on millet seeds.

**Figure 4:**
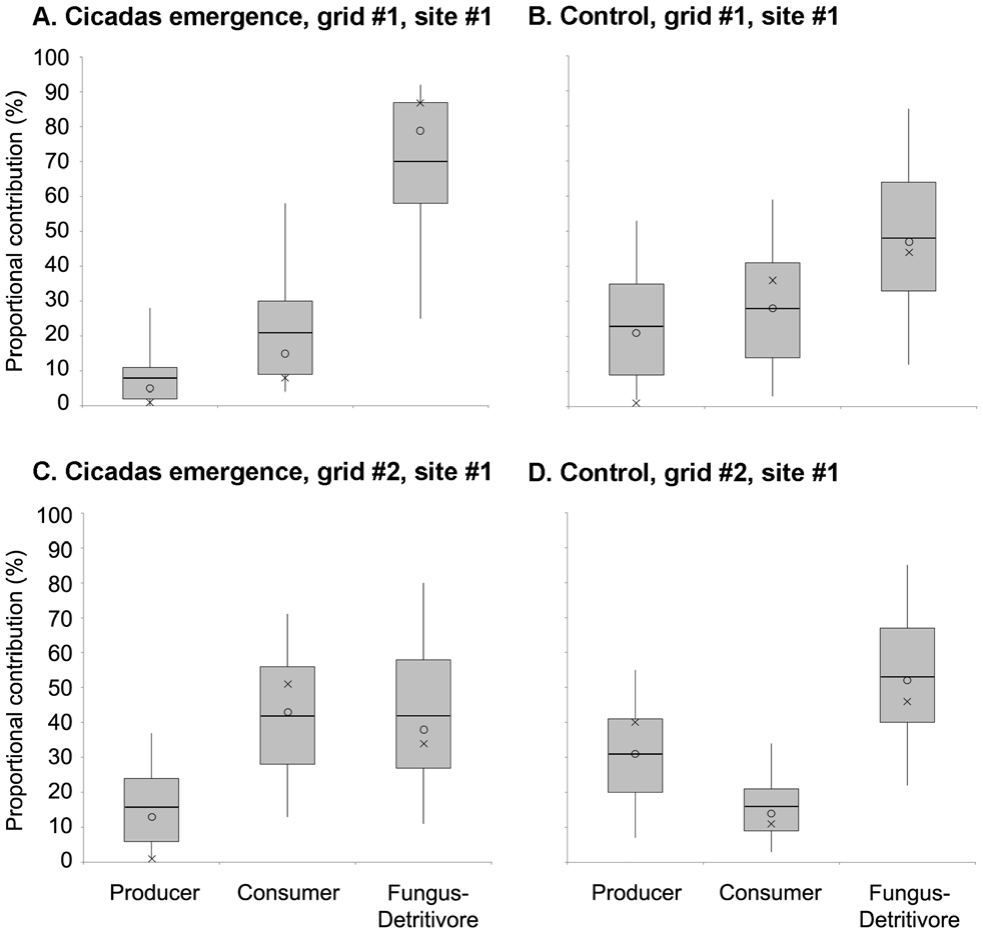
The producer, consumer, and fungus-detritivore contributions to the mouse diets in the cicadas emergence experiment. The cicadas emergence grids were plotted on the left and the control grids on the right for the first grid (A, B) and the second grid (C, D). The probability distributions of above-ground arthropods and the cicadas were combined to represent the consumer contributions in the cicadas emergence grids (A, C). The lines, circles, and crosses denote the means, medians, and modes. The boxes and whiskers denote the 25–75th and 5–95th percentiles, respectively.

In the cicadas emergence experiment, the consumer contributions decreased by 13% in the first grid but increased by 29% in the second grid during the emergence year (Fig. 4). The proportional contributions of the cicadas alone were 7% in the first grid (5–95th percentiles  = 1–33%) and 18% in the second (5–95th percentiles  = 2–39%), indicating that cicadas contribution was not as strong or consistent as millet seeds. The 7% and 18% of cicadas contributions also did not correspond to the changes in the consumer contributions, −13% and 29%, respectively. This suggests that dietary shifts of the mice in response to the cicadas pulse might be more complex than to the seed pulse.

## Discussion

Diet switching in generalist consumers can create complex trophic interactions [10]–[11]. When diet switching involves trophic shifts, it could also lead to broad food-web consequences [5]. In this study, we demonstrated that resource pulses can drive trophic shifts in an omnivorous consumer, the White-footed mouse. Specifically, the mice shifted their diets by 32% towards the producer trophic group when millet seeds were supplemented at both sites examined and by 29% towards the consumer trophic group when cicadas were available in at least one of the two grids examined. Following the general predictions of optimal foraging theory, opportunistic foragers are expected to switch their diets to the food resource that is the most abundant at the time. Therefore, it is not surprising that the mice switched to feeding on millet seeds in the seed addition grids, and cicadas in the cicadas emergence grids. However, our findings suggest that diet switching of a generalist consumer can be strong enough to allow them to play different functional roles in food webs under perturbations of resource pulses. Furthermore, many generalist consumers, including the White-footed mouse in current study, are known to increase their population densities following a resource pulse [29]–[36]. Therefore, the combined trophic shifts and numerical responses of generalist consumers could be a key factor determining how resource pulses might affect food web dynamics.

A recent meta-analysis by Yang et al. [48] suggested that resource pulses at producer level (i.e. plants) had larger consumer response magnitudes in terms of numerical responses than pulses at other trophic levels (e.g. heterotrophs). Our results suggest that the magnitudes of diet switching in the mice were larger for the seed pulse than for the cicadas pulse. Specifically, millet seeds comprised approximately one third of the mouse diets, whereas cicadas comprised less than one fifth. In addition, the directions of mouse trophic shifts were more consistent for the seed pulse than for the cicadas pulse. The producer contributions increased in both seed addition grids whereas the consumer contributions increased in one grid but decreased in the other. However, owing to several other differences between the seed and cicadas pulses (e.g. the timing, duration, and nutritional quality), we do not suggest that there is a stronger trophic shift of the mice to pulses at producer level than pulses at other trophic levels. Controlled experiments, preferably with several different types of resources at each trophic level to separate the effects of resource identity from their trophic-level membership, will be needed in order to draw general conclusions on the relationship between trophic shift magnitudes and the trophic levels of the pulses.

Conspecific density is an important factor determining foraging behaviors in the Deer mouse (*Peromyscus maniculatus*) [49], a closed relative to the White-footed mouse. Previous studies have shown that the White-footed mouse exhibit positive numerical responses to both seed and cicadas pulses, but with different temporal patterns [13], [29]–[36]. When a seed pulse occurs in the fall, it usually increases mouse density in the following summer [e.g. 34–35], but when it occurs in the spring, it does not seem to have any effects on mouse density [13]. One the other hand, when a cicadas pulse occurs in the spring, it increases mouse density immediately [13], [36], followed by a decrease in recruitment and increase in parasitism, resulting in a short-lived numerical response [13]. Given these patterns in the numerical responses of the mice to seed and cicadas pulses, we suppose the weaker and less consistent functional responses to the cicadas pulse observed here was partly a result of the rapid changes in the competition strength during the cicadas emergence event. The dietary and trophic shifts of the mice measured in this study had a short time frame. Therefore, the functional responses of the mice to the seed pulse were less likely to be influenced by changes in conspecific density.

Because fungi and detritivores derive their carbon and nitrogen from plant and animal matters, we can use the differences in δ^13^C and δ^15^N values of the plants or above-ground arthropods between grids and between sites as indicators of potential bias in applying generic isotope values for the fungus-detritivore group. The differences in the isotope values of the plants or arthropods between grids within the same site were relatively small, less than 2.2‰ for δ^13^C and 1.4‰ for δ^15^N, and were in the range of the variability within a trophic group (Table S2). The isotope values of the plants or arthropods between sites were relatively large. Specifically, site #2 can be as much as 3.1‰ lower than site #1 for δ^13^C and as much as 4.2‰ higher than site #1 for δ^15^N (Table S2). In order to investigate the consequences of applying the isotope values of the fungus-detritivore group for site #1 to site #2, we ran a different set of proportional contribution estimates for site #2 assuming the fungus-detritivore group at site #2 had a δ^13^C value of -25.7‰ and a δ^15^N value of 5.7‰ (δ^13^C_site#2_  =  δ^13^C_site#1_ −3.1‰, and δ^15^N_site#2_  =  δ^15^N_site#1_ +4.2‰). The results showed that the producer trophic group contributed 42% more to the mouse diets in the seed addition grid, and millet seeds alone contributed 42% to the mouse diets. Therefore, there was a 10% increase in the producer or millet contribution from the results based on the isotope values of the fungus-detritivore group at site #1. Nevertheless, the patterns remained the same: both sites had a consistent increase in the producer contribution with the seed pulse that could be explained by the increased consumption of millet seeds.

The inconsistent patterns in the cicadas emergence experiment were not likely a result of pre-existing dietary differences between the two grids. The differences in proportional contributions between the two grids in 2003 were less than 15% for any given trophic group (i.e. 10% differences for the producer, 14% for the consumer, and 5% for the fungus-detrivitore between the two grids; Fig. 4 B & D), which were more than doubled in 2004 during the cicadas emergence for two of the three trophic groups (i.e. 8% differences for the producer, 28% for the consumer, and 41% for the fungus-detrivitore between the two grids; Fig. 4 A & C). One possible explanation for the inconsistent patterns between the two grids is the spatial heterogeneity in cicadas density. Cicadas are known to aggregate on younger trees, possibly because the light environment of host trees had a positive effect on their oviposition density [50]. In fact, the percent canopy cover was measured at 50 locations (10 meters apart from one another) within each of the two grids in a separate study in 2002, and the results suggested that grid #1 had a slightly higher canopy cover than grid #2 (the 95% confidence intervals of the percent canopy cover: 92–96% for grid #1 and 85–94% for grid #2; Shaner, unpublished data). Therefore, it is likely that grid #1 had a lower cicadas density than grid #2, leading to the smaller cicadas contribution to the mouse diets in grid #1 (Fig. 4 A & C). This discussion also illustrates the importance of quantifying resource abundance in a natural experiment at a spatial scale appropriate for the consumer-resource interactions.

Stable isotopes are important tools for dietary studies at individual, population, and community levels [5], [18]. As demonstrated in current and previous studies, the Bayesian mixing model can produce robust estimates on dietary compositions. For example, Storm and Whitaker [51] reported that cicadas made up 25% of the stomach content of the White-footed mouse at a different site during the same 2004 emergence event, which is slightly higher than our estimates of 7% and 18%. It makes intuitive sense that stomach content analysis yielded a higher estimate, considering that cicadas exoskeleton are mostly indigestible to the mice but are highly detectable in the stomach. Interestingly, fungus-detritivore contributions to the mouse diets were estimated between 30–79% across all grids, which are much higher than previously reported based on stomach content analysis (<5% by volume or frequency) [25]–[26]. This discrepancy might be explained in part by a systematic bias of underestimating fungus-detritivore contributions in stomach content analysis due to the difficulties involved in identifying their fragments. In fact, fungi are believed to be a very important dietary source for many omnivorous mammals [52]–[54]. In a study using stable carbon isotopes, McIlwee and Johnson [55] demonstrated that the maximum contributions of fungus to the diets of two marsupial species (Northern Bettong *Bettongia tropica* and Rufous Bettong *Aepyprymnus rufescens*) were 25% and 67%, which are in agreement with our results on the White-footed mouse. Given the potential significance of fungi and detritivores as dietary sources for omnivorous mammals, it will be important to design future studies that specially address this trophic interaction.

Given the inherent difficulties in studying resource pulses, we presented here a practical approach that combines manipulative and natural experiments to show common patterns on dietary shifts in a generalist consumer responding to different types of resource pulses. This study also provides one of the few highly quantitative descriptions on the functional responses of a key consumer in forest food webs, which may help future studies to form specific predictions on changes in trophic interactions following resource pulses.

## Supporting Information

Table S1 **The isotope values and elemental concentrations of the plants, arthropods, and fungi included in the study.** Taxon-specific values of δ^13^C, δ^15^N, %C, and %N for the plants, arthropods, and fungi used in calculating dietary source values. “N” is the number of individual samples included for each taxon.
(DOC)

Table S2 **The isotope values and elemental concentrations of the dietary sources used in the mixing models.** Grid-specific values of δ^13^C, δ^15^N, %C, and %N for the fungi/detritivores, above-ground arthropods, plants, millet seeds, and cicadas. The mean value for each dietary source was calculated from taxon-specific values listed in Table S1.
(DOC)
