# Supplementary material for: Trophic Shifts of a Generalist Consumer in Response to Resource Pulses
Source: PLoS One. 2011 Mar 18;6(3):e17970. doi: 10.1371/journal.pone.0017970 (PMC3060883; doi:10.1371/journal.pone.0017970)
Supplement: Table S1 — The isotope values and elemental concentrations of the plants, arthropods, and fungi included in the study. Taxon-specific values of δ13C, δ15N, %C, and %N for the plants, arthropods, and fungi used in calculating dietary source values. “N” is the number of individual samples included for each taxon. (DOC) [file pone.0017970.s001.doc]

# Supporting Information

**Table S1. The isotope values and elemental concentrations of the plants, arthropods, and fungi included in the study.**

| Source | Taxa | δ^13^C ‰(±s.d.) | δ^15^N‰ (±s.d.) | %C (±s.d.) | %N (±s.d.) | N |
| --- | --- | --- | --- | --- | --- | --- |
| **Seed addition, grid #2, site #1** | | | | | | |
| Fungi/Detritivores | Diplopoda | -24.1 | 1.9 | 34.64 | 5.464 | 1 |
| Above-ground arthropods | Chilopoda | -23.6 | 3.9 | 46.86 | 13.96 | 1 |
|  | Hymenoptera: Formicidae | -27.6±0.4 | 6.0±0.1 | NA | NA | 2 |
|  | Orthoptera: Gryllidae | NA | NA | 48.46 | 11.66 | 1 |
|  | Coleoptera: Staphylinidae | -24.7 | 6.1 | 51.02 | 12.33 | 1 |
|  | Chlaenius sp. (Coleoptera: Carabidae) | -27.2 | 4.4 | 38.19 | 7.88 | 1 |
| Plants | Carya sp. | -29.1±1.9 | -1.9±0.6 | 38.36±7.79 | 2.35±0.50 | 2 |
|  | Celastrus orbiculatus | -31.7 | -1.4 | 58.46 | 2.25 | 1 |
|  | Celtis occidentalis | -29.6±0.1 | -3.4±0.8 | 41.08±12.02 | 3.37±0.11 | 2 |
|  | Quercus sp. | -28.6±0.3 | -2.6±0.6 | 49.20±0.18 | 2.68±0.57 | 2 |
| Millet seeds | Panicum miliaceum | -11.2±0.4 | 6.8±0.6 | 47.65±5.04 | 2.03±0.33 | 4 |
| **Seed control, grid #1, site #1** | | | | | | |
| Fungi/Detritivores | Diplopoda | -24.3 | 0.5 | 13.14 | 2.82 | 1 |
|  | Fungi | -21.0±0.2 | 1.9±1.8 | 33.81±3.92 | 2.66±1.32 | 2 |
| Above-ground arthropods | Coleoptera: Carabidae (Chlaenius sp.) | -28.7±2.4 | 5.6±2.5 | 55.93±10.58 | 9.12±3.87 | 3 |
|  | Coleoptera: Carabidae (Pterostichus sp.) | -30.2 | 1.3 | 62.36 | 6.41 | 1 |
|  | Hemiptera | -26.9 | 6.7 | 58.21 | 10.38 | 1 |
|  | Lepidoptera: Geometridae | -28.5 | 4.8 | 51.42 | 13.29 | 1 |
|  | Lepidoptera: Noctuid | -30.8 | 2.1 | 52.97 | 12.57 | 1 |
|  | Opiliones | -25.8±0.8 | 5.1±1.2 | 51.49±3.60 | 13.33±1.86 | 3 |
|  | Orthoptera: Gryllidae | -25.2 | 4.9 | 70.12 | 17.02 | 1 |
| Plants | Carya sp. | -30.7±1.9 | -2.1±0.9 | 48.63±4.09 | 2.35±0.01 | 2 |
|  | Celtis occidentalis | -28.6 | -0.5 | 29.58 | 3.50 | 1 |
|  | Cornus florida | -30.4 | -2.0 | 55.62 | 2.00 | 1 |
|  | Lindera benzoin | -30.7 | -2.3 | 52.35 | 3.66 | 1 |
|  | Lonicera sp. | -31.3±0.1 | -1.4±0.4 | 39.53±7.06 | 2.38±0.09 | 2 |
|  | Quercus sp. | -28.9±1.0 | -3.0±0.8 | 52.95±6.91 | 2.77±0.70 | 3 |
| **Seed addition, grid #2, site #2** | | | | | | |
| Above-ground arthropods | Araneae | -26.0 | 8.7 | 50.61 | 12.90 | 1 |
|  | Coleoptera: Carabidae (Chlaenius sp.) | -28.9±2.0 | 6.3±3.5 | 54.43±3.15 | 9.71±1.55 | 3 |
|  | Coleoptera: Nitidulidae | -23.0±0.1 | 7.8±0.1 | 52.51±0.69 | 11.44±0.11 | 2 |
|  | Coleoptera: Silphidae | -24.8±1.5 | 7.00±1.2 | 50.13±3.86 | 11.86±0.81 | 2 |
|  | Coleoptera: Staphylinidae | -25.6±0.2 | 8.9±0.1 | 52.08±1.62 | 11.68±0.41 | 2 |
|  | Hymenoptera: Formicidae | -24.8 | 6.6 | 49.52 | 11.76 | 1 |
|  | Opiliones | -26.2±0.4 | 7.6±1.0 | 49.64±1.46 | 11.46±1.51 | 2 |
|  | Orthoptera | -26.0 | 6.4 | 41.59 | 11.04 | 1 |
| Plants | Alliaria petiolata | -29.9 | 1.3 | NA | NA | 1 |
|  | Carya sp. | -29.1 | 3.1 | NA | NA | 1 |
|  | Celtis occidentalis | -29.4 | 3.1 | NA | NA | 1 |
|  | Geum sp. | -35.9 | 0.5 | 49.58 | 3.26 | 1 |
|  | Polygonum virginianum | -33.3 | 1.3 | 32.37 | 2.88 | 1 |
|  | Quercus sp. | -30.5 | 2.2 | 35.66 | 2.36 | 1 |
|  | Rhamnus cathartica | -32.4 | 1.7 | NA | NA | 1 |
| Millet seeds | Panicum miliaceum | -11.2±0.4 | 6.8±0.6 | 47.65±5.04 | 2.03±0.33 | 4 |
| **Seed control, grid #1, site #2** | | | | | | |
| Above-ground arthropods | Araneae | -25.6±0.9 | 6.8±0.6 | 49.73±2.26 | 13.02±1.83 | 2 |
|  | Coleoptera: Carabidae (Chlaenius sp.) | -26.2±2.1 | 8.7±0.7 | 52.13±2.82 | 12.92±1.73 | 3 |
|  | Coleoptera: Nitidulidae | -23.0±0.4 | 3.3±1.1 | 49.70±3.45 | 11.29±0.80 | 2 |
|  | Coleoptera: Staphylinidae | -26.4±1.9 | 7.7±0.7 | 52.04±0.57 | 11.53±0.04 | 2 |
|  | Hymenoptera: Formicidae | -25.5±2.1 | 6.1±1.0 | 48.08±2.60 | 12.06±0.79 | 3 |
|  | Opiliones | -27.5±0.5 | 6.6±1.1 | 48.91±5.85 | 9.86±0.93 | 2 |
| Plants | Berberis thunbergii | -31.7 | 2.1 | 58.44 | 2.66 | 1 |
|  | Celtis occidentalis | -29.9 | 1.5 | 50.27 | 3.50 | 1 |
|  | Duchesne indica | -35.6 | -0.2 | NA | NA | 1 |
|  | Duerella frutescens | -34.8 | 1.4 | 49.62 | 3.92 | 1 |
|  | Eleagnus sp. | -31.7 | 0.6 | 36.12 | 3.22 | 1 |
|  | Geum sp. | -33.9±0.6 | 1.6±0.6 | 36.59±6.88 | 3.20±0.12 | 2 |
|  | Lonicera tatarica | -30.5 | 0.1 | 39.60 | 2.13 | 1 |
|  | Polygonum cespitosum | -36.6 | 0.5 | 51.18 | 4.24 | 1 |
|  | Polygonum virginianum | -33.0 | -0.1 | 43.63 | 3.11 | 1 |
|  | Quercus sp. | -31.0 | -2.5 | 55.38 | 2.17 | 1 |
|  | Rhamnus cathartica | -31.6±1.1 | 0.2±0.8 | 55.39±5.13 | 2.49±0.39 | 2 |
| **Cicadas emergence, grid #1, site #1** | | | | | | |
| Fungi/Detritivores | Diplopoda | -24.3 | 0.5 | 13.14 | 2.82 | 1 |
|  | Fungi | -21.0±0.2 | 1.9±1.8 | 33.81±3.92 | 2.66±1.32 | 2 |
| Above-ground arthropods | Coleoptera: Carabidae (Chlaenius sp.) | -28.7±2.4 | 5.6±2.5 | 55.93±10.58 | 9.12±3.87 | 3 |
|  | Coleoptera: Carabidae (Pterostichus sp.) | -30.2 | 1.3 | 62.36 | 6.41 | 1 |
|  | Hemiptera | -26.9 | 6.7 | 58.21 | 10.38 | 1 |
|  | Lepidoptera: Geometridae | -28.5 | 4.8 | 51.42 | 13.29 | 1 |
|  | Lepidoptera: Noctuid | -30.8 | 2.1 | 52.97 | 12.57 | 1 |
|  | Opiliones | -25.8±0.8 | 5.1±1.2 | 51.49±3.60 | 13.33±1.86 | 3 |
|  | Orthoptera: Gryllidae | -25.2 | 4.9 | 70.12 | 17.02 | 1 |
| Cicadas | Magicicada spp. | -27.0±1.6 | -0.3±1.8 | 57.78±5.08 | 9.59±1.76 | 7 |
| Plants | Carya sp. | -30.7±1.9 | -2.1±0.9 | 48.63±4.09 | 2.35±0.01 | 2 |
|  | Celtis occidentalis | -28.6 | -0.5 | 29.58 | 3.50 | 1 |
|  | Cornus florida | -30.4 | -2.0 | 55.62 | 2.00 | 1 |
|  | Lindera benzoin | -30.7 | -2.3 | 52.35 | 3.66 | 1 |
|  | Lonicera sp. | -31.3±0.1 | -1.4±0.4 | 39.53±7.06 | 2.38±0.09 | 2 |
|  | Quercus sp. | -28.9±1.0 | -3.0±0.8 | 52.95±6.91 | 2.77±0.70 | 3 |
| **Cicadas control, grid #1, site #1** | | | | | | |
| Fungi/Detritivores | Diplopoda | -24.3 | 0.5 | 13.14 | 2.82 | 1 |
|  | Fungi | -21.0±0.2 | 1.9±1.8 | 33.81±3.92 | 2.66±1.32 | 2 |
| Above-ground arthropods | Coleoptera: Carabidae (Chlaenius sp.) | -28.7±2.4 | 5.6±2.5 | 55.93±10.58 | 9.12±3.87 | 3 |
|  | Coleoptera: Carabidae (Pterostichus sp.) | -30.2 | 1.3 | 62.36 | 6.41 | 1 |
|  | Hemiptera | -26.9 | 6.7 | 58.21 | 10.38 | 1 |
|  | Lepidoptera: Geometridae | -28.5 | 4.8 | 51.42 | 13.29 | 1 |
|  | Lepidoptera: Noctuid | -30.8 | 2.1 | 52.97 | 12.57 | 1 |
|  | Opiliones | -25.8±0.8 | 5.1±1.2 | 51.49±3.60 | 13.33±1.86 | 3 |
|  | Orthoptera: Gryllidae | -25.2 | 4.9 | 70.12 | 17.02 | 1 |
| Plants | Carya sp. | -30.7±1.9 | -2.1±0.9 | 48.63±4.09 | 2.35±0.01 | 2 |
|  | Celtis occidentalis | -28.6 | -0.5 | 29.58 | 3.50 | 1 |
|  | Cornus florida | -30.4 | -2.0 | 55.62 | 2.00 | 1 |
|  | Lindera benzoin | -30.7 | -2.3 | 52.35 | 3.66 | 1 |
|  | Lonicera sp. | -31.3±0.1 | -1.4±0.4 | 39.53±7.06 | 2.38±0.09 | 2 |
|  | Quercus sp. | -28.9±1.0 | -3.0±0.8 | 52.95±6.91 | 2.77±0.70 | 3 |
| **Cicadas emergence, grid #2, site #1** | | | | | | |
| Fungi/Detritivores | Diplopoda | -24.1 | 1.9 | 34.64 | 5.464 | 1 |
| Above-ground arthropods | Chilopoda | -23.6 | 3.9 | 46.86 | 13.96 | 1 |
|  | Hymenoptera: Formicidae | -27.6±0.4 | 6.0±0.1 | NA | NA | 2 |
|  | Orthoptera: Gryllidae | NA | NA | 48.46 | 11.66 | 1 |
|  | Coleoptera: Staphylinidae | -24.7 | 6.1 | 51.02 | 12.33 | 1 |
|  | Chlaenius sp. (Coleoptera: Carabidae) | -27.2 | 4.4 | 38.19 | 7.88 | 1 |
| Cicadas | Magicicada spp. | -27.0±1.6 | -0.3±1.8 | 57.78±5.08 | 9.59±1.76 | 7 |
| Plants | Carya sp. | -29.1±1.9 | -1.9±0.6 | 38.36±7.79 | 2.35±0.50 | 2 |
|  | Celastrus orbiculatus | -31.7 | -1.4 | 58.46 | 2.25 | 1 |
|  | Celtis occidentalis | -29.6±0.1 | -3.4±0.8 | 41.08±12.02 | 3.37±0.11 | 2 |
|  | Quercus sp. | -28.6±0.3 | -2.6±0.6 | 49.20±0.18 | 2.68±0.57 | 2 |
| **Cicadas control, grid #2, site #1** | | | | | | |
| Fungi/Detritivores | Diplopoda | -24.1 | 1.9 | 34.64 | 5.464 | 1 |
| Above-ground arthropods | Chilopoda | -23.6 | 3.9 | 46.86 | 13.96 | 1 |
|  | Hymenoptera: Formicidae | -27.6±0.4 | 6.0±0.1 | NA | NA | 2 |
|  | Orthoptera: Gryllidae | NA | NA | 48.46 | 11.66 | 1 |
|  | Coleoptera: Staphylinidae | -24.7 | 6.1 | 51.02 | 12.33 | 1 |
|  | Chlaenius sp. (Coleoptera: Carabidae) | -27.2 | 4.4 | 38.19 | 7.88 | 1 |
| Plants | Carya sp. | -29.1±1.9 | -1.9±0.6 | 38.36±7.79 | 2.35±0.50 | 2 |
|  | Celastrus orbiculatus | -31.7 | -1.4 | 58.46 | 2.25 | 1 |
|  | Celtis occidentalis | -29.6±0.1 | -3.4±0.8 | 41.08±12.02 | 3.37±0.11 | 2 |
|  | Quercus sp. | -28.6±0.3 | -2.6±0.6 | 49.20±0.18 | 2.68±0.57 | 2 |
