# Supplementary material for: Trophic Shifts of a Generalist Consumer in Response to Resource Pulses
Source: PLoS One. 2011 Mar 18;6(3):e17970. doi: 10.1371/journal.pone.0017970 (PMC3060883; doi:10.1371/journal.pone.0017970)
Supplement: Table S2 — The isotope values and elemental concentrations of the dietary sources used in the mixing models. Grid-specific values of δ13C, δ15N, %C, and %N for the fungi/detritivores, above-ground arthropods, plants, millet seeds, and cicadas. The mean value for each dietary source was calculated from taxon-specific values listed in Table S1. (DOC) [file pone.0017970.s002.doc]

# Supporting Information

**Table S2. The isotope values and elemental concentrations of the dietary sources used in the mixing models.**

| **Source** | δ^13^C‰ (±s.d.)‡ | δ^15^N‰ (±s.d.)‡ | %C (±s.d.)‡ | %N (±s.d.)‡ | **Number of Taxa**§ |
| --- | --- | --- | --- | --- | --- |
| **Seed addition, grid #2, site #1** |  |  |  |  |  |
| Fungi/Detritivores¶ | -22.6±2.3 | 1.5±0.5 | 28.85±7.01 | 3.40±1.05 | 2 |
| Above-ground arthropods | -25.8±1.9 | 5.1±1.1 | 46.13±5.57 | 11.46±2.57 | 4 |
| Plants | -29.7±1.4 | -2.3±0.9 | 46.77±9.05 | 2.66±0.51 | 4 |
| **Millet seeds** | -11.2±0.4 | 6.8±0.6 | 47.65±5.04 | 2.03±0.33 | 1 |
| **Seed control, grid #1, site #1** |  |  |  |  |  |
| Fungi/Detritivores¶ | -22.6±2.3 | 1.5±0.5 | 28.85±7.01 | 3.40±1.05 | 2 |
| Above-ground arthropods | -28.0±2.1 | 4.4±1.9 | 57.50±6.82 | 11.73±3.43 | 7 |
| Plants | -30.1±1.1 | -1.9±0.9 | 46.44±9.98 | 2.78±0.67 | 6 |
| **Seed addition, grid #2, site #2** |  |  |  |  |  |
| Fungi/Detritivores¶ | -22.6±2.3 | 1.5±0.5 | 28.85±7.01 | 3.40±1.05 | 2 |
| Above-ground arthropods | -25.6±1.7 | 7.4±1.0 | 50.06±3.81 | 11.48±0.89 | 8 |
| Plants | -31.5±2.5 | 1.9±1.0 | 39.20±9.14 | 2.83±0.45 | 7 |
| **Millet seeds** | -11.2±0.4 | 6.8±0.6 | 47.65±5.04 | 2.03±0.33 | 1 |
| **Seed control, grid #1, site #2** |  |  |  |  |  |
| Fungi/Detritivores¶ | -22.6±2.3 | 1.5±0.5 | 28.85±7.01 | 3.40±1.05 | 2 |
| Above-ground arthropods | -25.7±1.5 | 6.5±1.8 | 50.10±1.65 | 11.78±1.18 | 6 |
| Plants | -32.8±2.2 | 0.5±1.3 | 47.62±8.14 | 3.06±0.71 | 11 |
| **Cicadas emergence, grid #1, site #1** |  |  |  |  |  |
| Fungi/Detritivores¶ | -22.6±2.3 | 1.5±0.5 | 28.85±7.01 | 3.40±1.05 | 2 |
| Above-ground arthropods | -28.0±2.1 | 4.4±1.9 | 57.50±6.82 | 11.73±3.43 | 7 |
| Plants | -30.1±1.1 | -1.9±0.9 | 46.44±9.98 | 2.78±0.67 | 6 |
| **Cicadas** | -27.0±1.6 | -0.3±1.8 | 57.78±5.08 | 9.59±1.76 | 1 |
| **Cicadas control, grid #1, site #1** |  |  |  |  |  |
| Fungi/Detritivores¶ | -22.6±2.3 | 1.5±0.5 | 28.85±7.01 | 3.40±1.05 | 2 |
| Above-ground arthropods | -28.0±2.1 | 4.4±1.9 | 57.50±6.82 | 11.73±3.43 | 7 |
| Plants | -30.1±1.1 | -1.9±0.9 | 46.44±9.98 | 2.78±0.67 | 6 |
| **Cicadas emergence, grid #2, site #1** |  |  |  |  |  |
| Fungi/Detritivores¶ | -22.6±2.3 | 1.5±0.5 | 28.85±7.01 | 3.40±1.05 | 2 |
| Above-ground arthropods | -25.8±1.9 | 5.1±1.1 | 46.13±5.57 | 11.46±2.57 | 4 |
| Plants | -29.7±1.4 | -2.3±0.9 | 46.77±9.05 | 2.66±0.51 | 4 |
| **Cicadas** | -27.0±1.6 | -0.3±1.8 | 57.78±5.08 | 9.59±1.76 | 1 |
| **Cicadas control, grid #2, site #1** |  |  |  |  |  |
| Fungi/Detritivores¶ | -22.6±2.3 | 1.5±0.5 | 28.85±7.01 | 3.40±1.05 | 2 |
| Above-ground arthropods | -25.8±1.9 | 5.1±1.1 | 46.13±5.57 | 11.46±2.57 | 4 |
| Plants | -29.7±1.4 | -2.3±0.9 | 46.77±9.05 | 2.66±0.51 | 4 |

**‡ The standard deviations (s.d.) of source isotope values were incorporated in the Bayesian mixing model to reflect the variability associated with source isotope values. However, the variability associated with elemental concentrations of dietary sources has not yet been implicated in SIAR.**

**§ For the full list of taxon-specific isotope and elemental concentration values, see Table S1.**

**¶ The fungus and detritivore samples were only available from one site (site #1; Table S1). Therefore, the isotope and elemental concentration values of the available fungus and detritivore samples were applied to all grids. Because there were substantial differences between sites in the isotope values of the plants and above-ground arthropods, another set of the isotope values for the fungus-detritivore group, adjusted for the maximum differences between sites observed for either the plants and above-ground arthropods, were applied to site #2 (adjusted fungus-detritivore group for site #2: δ^13^C_site#2_ = δ^13^C_site#1_ -3.1‰ = -25.7‰ and δ^15^N_site#2_ = δ^15^N_site#1_ +4.2‰ = 5.7‰). This resulted in a 10% increase in the producer or millet contribution from the results based on the isotope values of the fungus-detritivore group at site #1. Nevertheless, the patterns remained the same: both sites had a consistent increase in the producer contribution with the seed pulse that could be explained by the increased consumption of millet seeds.**
